# Risk factors of acute kidney injury after orthotopic liver transplantation in China

**DOI:** 10.1038/srep41555

**Published:** 2017-01-30

**Authors:** Yin Zongyi, Li Baifeng, Zou Funian, Li Hao, Wang Xin

**Affiliations:** 1 Department of hepatobiliary surgery and organ transplantation, the First Hospital of China Medical University, Shenyang 110001, China

## Abstract

In this study, we determined the risk factors for acute kidney injury (AKI) following orthotopic liver transplantation (OLT) in China. We collected 5074 donation after cardiac death (DCD) OLT recipients who underwent surgery between January 1, 2010, and December 31, 2015, in 86 academic hospitals or transplant centers in China. Univariate and multivariate analyses were used to investigate the criticality of donor, graft, or recipient variables in the development of post-OLT AKI. In all, 4482 patients were included (median age, 49.31 years). Post-OLT AKI occurred in 3.97% patients, and 73.6% of all OLT patients were male. The 1- and 5-year cumulative survival rates (CSRs) of the AKI group were 33.95% and 25.24%, respectively, compared with 86.34% and 70.05%, respectively, of the non-AKI group (P < 0.001). The independent risk factors for post-OLT AKI were blood loss, cold ischemia time, warm ischemia time, preoperative serum creatinine, the treatment period with dopamine, overexposure to calcineurin inhibitor, and combined mycophenolate mofetil use (P < 0.05). These had a high prediction accuracy for post-OLT AKI (area under the curve [AUC] = 0.740).

Acute kidney injury (AKI) is a common complication of orthotopic liver transplantation (OLT) and is a major cause of mortality and morbidity^1^,^2^,^3^,^4^,^5^. The incidence rate of AKI after OLT ranks from 5 to 94%^1^,^3^,^6^ and approximately 8–17% need renal replacement therapy (RRT)^3^,^7^. The Risk, Injury, Failure, Loss, and End-stage Renal Dysfunction (RIFLE) classification was set up for stratifying the severity of early AKI by the Acute Dialysis Quality Initiative (ADQI) workgroup^8^.

Various factors are associated with the occurrence of AKI, and some depend on the preoperative recipient’s condition, and others stem from intraoperative vascular and metabolic dynamics and postoperative complications^3^,^7^,^9^,^10^,^11^. Recipients may have had intrinsic renal disease, induced by obstructive uropathy hypotension^11^, cerebrovascular diseases^4^, diabetes mellitus^11^, or hepatorenal syndrome (HS)^12^ before undergoing liver transplantation. Acute tubular necrosis (ATN) and glomerulonephritis mainly develop in patients with nephrotoxic drug use, cirrhosis, and IgA nephropathy, all of them thought as signs of AKI^13^. Besides, graft size and donor age^14^, low prothrombin activity, and high pre-transplantation serum creatinine (SCr) level^15^ were also important identifiable risk factors of AKI after OLT. In addition to low cryoprecipitate transfusion^16^ and low intraoperative volume of blood transfusion^17^, immunosuppressive therapy of low nephrotoxic potential, and careful operative technique^18^ were recommended to diminish the risk for AKI after OLT. Meanwhile, some investigations revealed that postoperative infection^19^, RRT induction^14^ were independent risk factors of AKI complicated to OLT. However, some risk factors, which may cause AKI after OLT, are still controversial or even contradicting in different investigations^6^,^11^,^16^,^17^,^20^,^21^,^22^,^23^,^24^,^25^.

China is a big country for organ transplantation, which began its first clinical liver transplantation (LT) in 1977, and the cumulative number of LT is at least 30,000^26^,^27^. However, no national laws were framed for the oversight of China’s transplantation system until 2007^28^, and more than 90% of transplanted organs were obtained from executed prisoners^29^, an approach that violated medical ethics and was illegal. Thus, international scholars and journals boycotted to accept and publish the results of organ transplantation from China^30^. To date, limited data are available on patients with AKI who underwent OLT in China.

We attempted to explore in our study the independent risk factors of AKI after OLT with analysis of legal multicenter and large samples, and provide reliable data to recognize, prevent, and treat AKI after OLT in China.

## Results

**Figure 1:**
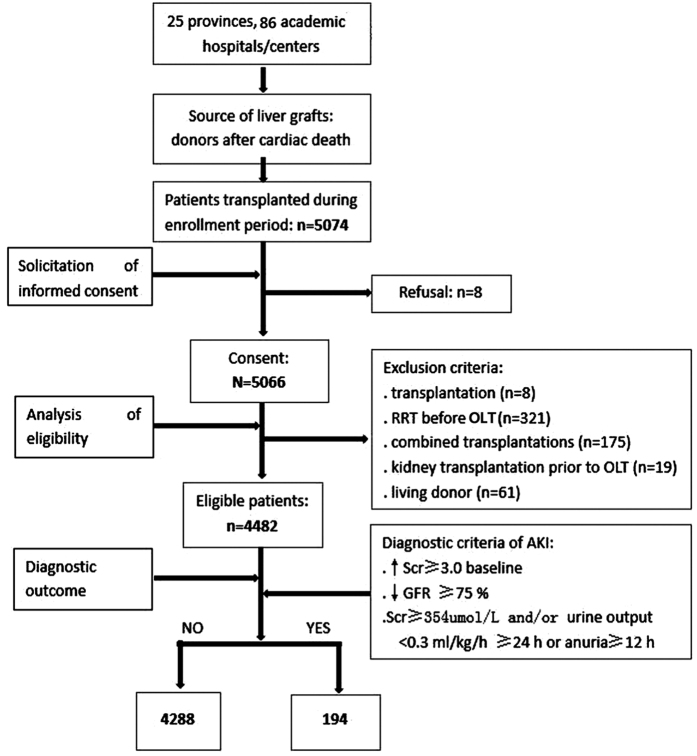
Study profile. RRT = renal dialysis treatment; OLT = orthotopic liver transplantation.

As shown in Fig. 1, 5074 adult recipients underwent OLT during our enrollment period, 584 patients were excluded (8, 321, 175, 19, and 61 cases for re-transplantation, RRT before OLT, combined transplantations, kidney transplantation before OLT, and living donor, respectively), and 8 patients refused to sign the informed consent. Finally, 4482 patients were included in our investigation, and their clinical and biochemical data were analyzed.

### Donor characteristics based on AKI and non-AKI after OLT

Some reports^14^,^31^ have confirmed that certain factors derived from donors influence the OLT prognosis. Herein, we attempted to explore the association between donor factors and occurrence of post-OLT AKI. The mean age in the non-AKI and AKI groups was 37.92 years (22.63, 47.63) and 39 years (24, 47.17), respectively (Table 1). Male patients accounted for 73.23% (3140/4288) and 79.90% (155/194) in the non-AKI and AKI groups, respectively. No significant differences were found in age, sex, donor blood type, and past medical history, but the China classification of DCD and cause of death showed a remarkable difference between groups (P = 0.005 and P = 0.007, respectively). Moreover, we analyzed biochemical markers of donor blood. No significant difference was found between groups regarding K^+^, ALP, total bilirubin, GGT, and ALT. However, the AKI group showed a higher concentration of Na^+^ (153.15 vs 144 mmol/L, P = 0.008), BUN (9.78 vs 6.83 mmol/L, P = 0.001), blood sugar (8.5 vs 7.65 mmol/L, P = 0.039), SCr (103.5 vs 79 μmol/L, P < 0.001), and AST (61 vs 50.4 U/L, P = 0.011), and a lower albumin level (30.6 vs 33.38 g/L, P = 0.003) than the non-AKI group.

### Demographic and clinic data of recipients with or without AKI after OLT

The mean age of the 4482 enrolled patients was 49.31 years, and male patients accounted for 73.56% (3591/4882). AKI after OLT occurred in 3.97% (194/4882) recipients. The age in the AKI group was slightly increased than that in the non-AKI group (50.5 vs 48.4 years, P = 0.056). No significant difference was found regarding sex and BMI between these two groups (Table 2).

Tacrolimus and cyclosporine with or without MMF were administered to recipients on day 0 after surgery. Induction therapy using basiliximab, rabbit antithymocyte globulin (rATG) or anti-Tac mAb, sometimes combined with steroid drugs, was administered to 2151 (48%) patients. A steroid-based therapy was administered to 3675 (82%) patients (data not shown).

No difference was observed in the preoperative data analysis (Table 2) between the two groups regarding hepatitis B, preoperative dialysis, total bilirubin, INR, MELD score, Child–Pugh grade, and albumin, which were paradoxical with the findings in Naik *et al*.^11^ and Yuan *et al*.’s^19^ studies. Furthermore, we found that a remarkable association was found between the AKI and non-AKI groups regarding blood type compatibility (90.21% vs 87.62%, P = 0.004), hepatocarcinoma-related disease (P < 0.001), cirrhosis (71.13% vs 79.55%, P = 0.005), hepatic failure (18.04% vs 7.25%, P < 0.001), and SCr (85 vs 74.6 μmol/L, P = 0.003).

Wenger *et al*.^25^ have confirmed that many intraoperative data like duration of surgery and blood transfusion volume were independent factors contributing to post-OLT AKI. Here, we also observed a significant difference between groups in cold ischemia time (7 vs 6.17 h, P = 0.043), warm ischemia time (5 vs 5 min, P < 0.001), intraoperative blood loss (2500 vs 1600 mL, P < 0.001), red blood cells (2000 vs 1600 mL, P < 0.001), autologous blood cell transfusion (1225 vs 800 mL, P = 0.015), fresh frozen plasma (1850 vs 1450 mL, P < 0.001), and total intravenous infusion (6611.5 vs 5500 mL, P < 0.001). Furthermore, patients with post-OLT AKI had a greater need for noradrenaline (P < 0.001) and dobutamine (P < 0.001). However, no difference was found between groups regarding duration of surgery, whole blood, cell saver, and platelet (Table 3).

We attempted to postoperatively analyze the association between post-OLT complications and occurrence of AKI. Interestingly, a significant difference was found between the AKI and non-AKI groups regarding intraperitoneal hemorrhage (32.99% vs 4.52%, P < 0.001), vascular complications (8.76% vs 3.54%, P < 0.001), abnormal graft function (28.35% vs 1.66%, P < 0.001), postoperative infection (44.85% vs 15.79%, P < 0.001), diabetes (21.65% vs 12.52%, P < 0.001), hypertension (6.19% vs 3.36%, P = 0.036), new-onset diabetes (16.49% vs 10.03%, P = 0.004), pleural effusion (48.97% vs 23.06%, P < 0.001), pneumonedema (10.31% vs 0.93%, P < 0.001), and intra-abdominal abscess (32.99% vs 11.22%, P < 0.001). In addition, hospital stay length and follow-up duration of the AKI group were remarkably shorter compared with those in the non-AKI group (16 vs 25 days, P < 0.001; 0.72 vs 3.82 months, P < 0.001, respectively) (Table 4).

Besides, some previous studies^24^,^32^ showed that immunosuppressive therapy can result in post-OLT AKI. Here, we also attempted to analyze the association between post-OLT AKI and immunosuppressive agent use. The results show that a significant difference was found between the AKI and non-AKI groups regarding initial induction of CNI with tacrolimus (75.02% vs. 62.51%, P = 0.015), induction of CNI with cyclosporine (13.04% vs. 9.11%, P = 0.015), and mean CNI trough level for tacrolimus (10.3 vs. 9.2 ng/mL, P = 0.008). The overexposure rate to CNI was significantly higher in the AKI group (P < 0.001). With regard to MMF use, a remarkable difference was found between both groups (71.08% vs. 42.10%, P < 0.001).

### Cumulative survival rates of recipients and grafts with or without post-OLT AKI

**Figure 2:**
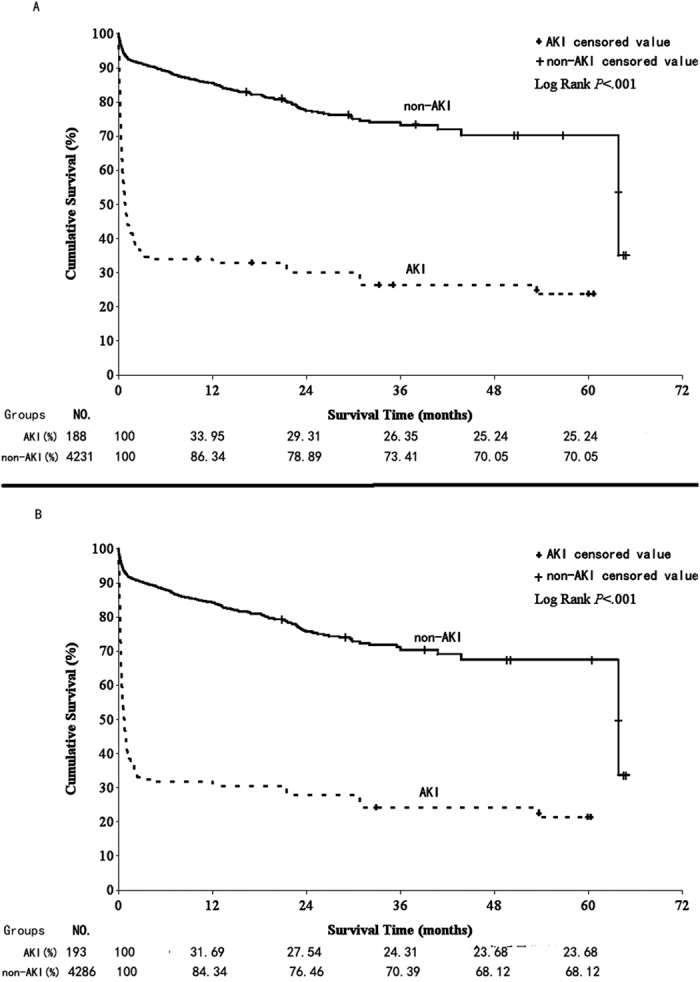
Kaplan-Meier Curves for (**A**) the recipients and (**B**) the grafts.

During long-term follow-up, we found that the 1- and 5-year cumulative survival rates (CSR) were 83.34% and 68.14%, respectively, in all eligible recipients. However, the CSR of the AKI group was significantly lower than that of the non-AKI group. The 1- and 5-year CSRs were 33.95% and 25.24% in the AKI group compared with 86.34% and 70.05% in the non-AKI group, respectively (P < 0.001) (Fig. 2A). Moreover, the 1- and 5-year CSR between the AKI and non-AKI groups were 31.69% vs 84.34% and 23.68% vs 68.12% (P < 0.001), respectively with respect to the grafts (Fig. 2B). In fact, approximately two thirds of recipients or grafts died within the first month after OLT in the AKI group. With regard to the recipients, the first-month CSR was 38% 95% in the AKI and non-AKI groups, respectively.

### Multivariate analysis of risk factors associated with post-OLT AKI

A stepwise logistic regression model was constructed based on significant univariate analysis data to find the independent risk factors for post-OLT AKI. After multivariable risk adjustment for potential confounding factors (Table 5), cold ischemia time (OR, 1.061; 95% CI, 1.032–1.090; P < 0.001), warm ischemia time (OR, 1.028; 95% CI, 1.011–1.046; P = 0.001), blood loss (OR, 230; 95% CI, 1.001–1.451; P < 0.001), SCr (OR, 1.352; 95% CI, 1.181–1.763; P < 0.001), treatment period with dopamine (OR, 1.854; 95% CI, 1.425–2.281; P < 0.001), overexposure to CNI (OR, 2.841; 95% CI, 1.762–5.360; P < 0.001), and combined MMF use were still positively correlated with post-OLT AKI (P = 0.023).

**Figure 3:**
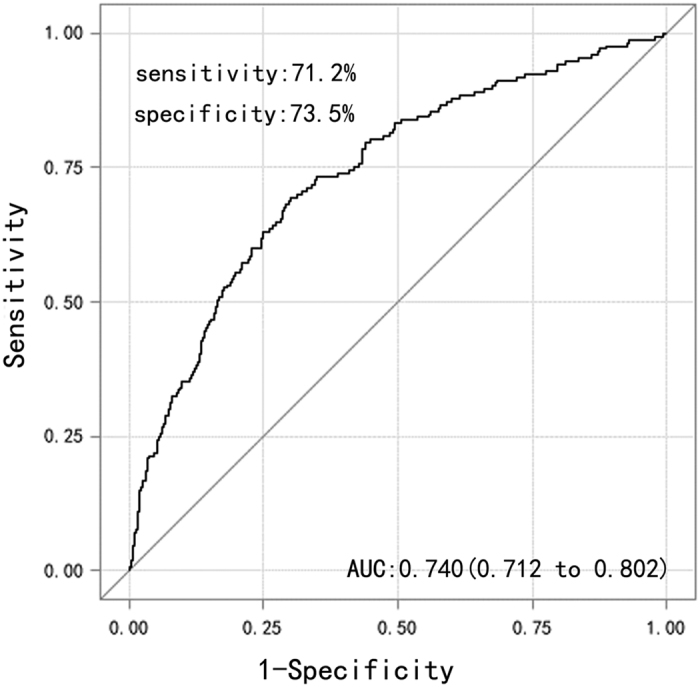
Receiver operating characteristic (AUC) cure of the risk factors for the occurrence post-OLT AKI.

Moreover, the predictive ability of these seven risk factors for post-OLT AKI was evaluated by using ROC analysis. The AUC for this model was 0.740 (95% CI, 0.712–0.802; sensitivity = 71.2%; specificity = 73.5%; Fig. 3). Furthermore, the H–L test was conducted for logistic regression, and no evidence showed lack of fit (P = 0.142; data no shown). In summary, all these results indicated that the seven factors were independent risk factors the occurrence of post-OLT AKI.

## Discussion

AKI is a common and severe complication after OLT and seriously affects the prognosis of OLT patients^2^,^33^,^34^. Because the major proportion of transplanted organs in China were obtained from illegal and unethical approaches, such as convicted prisoners and human organ traders, before 2015, limited information regarding organ transplantation, including post-OLT complications, was reported in public or international journals^29^,^30^. Herein, we reported the incidence rate of post-OLT AKI and analyzed its possible risk factors in China. In addition, this study reported this complication with analysis of multicenter and large samples in China.

In the study, we discovered that the incidence rate of post-OLT AKI was 3.97%, which was less than the 11.1–90% reported in previous studies^1^,^3^,^6^,^35^,^36^,^37^, and similar to the finding of Kirnap *et al*.’s study (5%)^20^. This relatively low occurrence rate benefited from the development of transplantation surgery and application of precautionary measures to prevent complications after OLT^17^,^18^,^22^,^38^. Compared with the occurrence rate approximately 10 years prior^10^,^34^,^39^,^40^, it decreased gradually in recent years^20^,^35^, and our result is consistent with this tendency. In addition, using the difference diagnosis criteria of AKI also could be a reason for the reporting of different rates. One study have reported a 51.5% occurrence rate using the definition of SCr > 1.5 mg/dL^41^, and 17% was reported by using SCr > 2 mg/dL in another study^11^. In the present study, we defined AKI based on the RIFLE classification, such as that used in Liu *et al*.’s study^16^. This comprehensive definition may account for the relatively low incidence rate of AKI observed.

The 1- and 5-year survival rates were speculated as important marks for OLT prognosis. Previous studies have demonstrated that post-OLT AKI was associated with an increasing eight-fold mortality risk^42^, prolonged stay in the intensive care unit, and augmented hospital costs^13^. In our investigation, the 1- and 5-year CSRs were 83.34% and 68.14%, respectively, in all eligible recipients, but had a significant difference in subgroups (AKI group, 33.95% and 25.24%; non-AKI group, 86.34% and 70.05%; P < 0.05). This result was similar to that of Nonthasoot *et al*.’s study in Thailand (1- and 5-year CSRs: 85% and 69%)^36^, but remarkably differed from that of Kirnap *et al*.’s study in Turkey (1-year CSR in the AKI and non-AKI groups was 82% and 89%, respectively)^20^ and O’Riordan *et al*.’s study in Denmark (1-year CSR in the AKI group was 47.5%)^35^. The reason for these differences may be related to economic level and health care quality (Denmark and Turkey are developed countries, whereas China and Thailand are low-income countries). Studies have demonstrated that patients with AKI could have a recovery rate of 97% and result in a good prognosis with appropriate treatments^13^,^43^. In addition, with regard to the recipients, the first-month CSR was 38% in AKI group, which was lower than that in the non-AKI group. The exact reasons were unclear, and it may be related to poor management of AKI recipients^44^.

Donor factors were considered important in organ transplantation and directly affected survival rates of recipients^14^,^31^,^45^,^46^,^47^. Here, our results indicated that China classification of DCD, donor cause of death, and blood constituent have shown a remarkable difference between the AKI and non-AKI groups. However, no evidence supports in proving that the donor factors included in our study were independent risk factors associated with post-OLT AKI. Unfortunately, other donor factors, such as BMI^47^, graft weight and size^24^, and pre-arrest SCr^31^ confirmed to be related to AKI after OLT in previous studies, were not registered in our database, and feasible comparison cannot be conducted.

Furthermore, we analyzed the relationship between the features of recipient and occurrence of post-OLT AKI. The result indicated that males account for 73.6% of all recipients, which may be attributed to a higher occurrence rate of hepatocarcinoma (male:female = 3:1) in males^48^.

Besides, in preoperative factor analysis, we found that blood type compatibility, hepatocarcinoma-related diseases, cirrhosis, hepatic failure, and SC were significantly different between the AKI and non-AKI groups, which were correlated with previous studies^11^,^16^,^19^,^35^,^49^,^50^. However, in contrast with Utsumi *et al*.’s^24^ and Yuan *et al*.’s^19^ studies, no evidence supports that higher MELD score and Child–Pugh grade were related to post-OLT AKI in our investigation and Faenza *et al*.’s^49^. The reasons may be related to (1) the inclusion of patients in our study with relatively low SCr level (we excluded patients who are on RRT as they are not “at risk” for post-operative renal injury); (2) the MELD score lacking systematic observed indicators, such as ascites, blood loss, hepatic encephalopathy, etc., but not just SCr, INR, total bilirubin; hence, estimating the prognosis of patients who have no preoperative AKI was not suitable^51^; and (3) the Sequential Organ Failure Assessment score having the best discriminatory power for prediction of short-term mortality after OLT as indicated by Elsayed *et al*.^52^. In addition, hypoalbuminemia in Cabezuelo *et al*.’s study^3^ showed a significant difference between two groups, but it was contradictory to our results. The exact reasons were still unclear, it might be probably due to the following: (1) the small sample size in Cabezuelo *et al*.’s study (184 patients); (2) exclusion of the preoperative RRT population in our study, but not in their study; and (3) the development of parenteral alimentation, which can regulate serum protein level in recent years.

In the present study, we found an obvious difference between the AKI and non-AKI groups, regarding intraoperative blood loss, transfusion of blood products, cold or warm ischemia time, and total volume of intravenous infusion. These findings were consistent with previous studies^6^,^16^,^18^,^19^,^24^,^25^,^49^. However, regarding the duration of surgery, no significant difference was found between groups in our results, but some studies^17^,^32^,^53^ inferred that this can be attributed to post-OLT AKI. In fact, the duration of surgery is influenced by many factors. Only those factors, such as prolonged blood loss, long-standing hypoxemia, and so on, can result in the instability of systemic hemodynamics and kidney injury after surgery. A good explanation why venovenous bypass was not related to post-OLT AKI was because it can regulate the stability of systemic hemodynamics.

In addition, the changes of hemodynamic factors during surgery have an effect on the development of post-OLT AKI. Recipients requiring vasoactive medications usually had lower MAP and systolic arterial pressure during the anhepatic phase, and lower cardiac index during the post-anhepatic phase. Renal blood flow reduction could affect postoperative renal function through different mechanisms. Dealing with the changes of OLT hemodynamics was difficult. Bilbao *et al*.^9^ confirmed that patients with more vasoactive drugs use have a higher probability of requiring post-OLT RRT. Our results that post-OLT AKI group had a greater need for adrenergic agonist drugs and blood products than non-AKI group during and after OLT were in line with these studies^3^,^54^,^55^. Noradrenaline, dobutamine, and dopamine all showed a strong correlation with the occurrence of post-OLT AKI, and the treatment period with dopamine (>6 days) was an independent factor for post-OLT AKI. Thus, we believe that patients with greater hemodynamic instability are more susceptible to developing AKI^3^.

Moreover, we also analyzed the association between post-OLT AKI and other postoperative complications, and results indicated significant difference between the two groups regarding postoperative infection, diabetes, and hypertension, which were similar to previous studies^6^,^19^,^24^,^35^. The possible reason was these complications could increase the burden of renal function through different mechanisms in early post-OLT^24^. Furthermore, we compared the hospital stay length and follow-up duration in the AKI and non-AKI groups. Surprisingly, compared with those without AKI, the mean length of hospital stay was significantly lower for those with AKI. The reasons may be that (1) more patients with AKI died within the first 2 weeks post-transplantation; (2) patients with AKI were transferred to nephrology department or other kidney disease centers for RRT, because some organ transplant centers, in China, had not equipment for RRT; (3) patients with AKI could not afford for the cost of RRT and left hospitals reluctantly within two weeks post-transplantation, as the health insurance, in China, did not cover the cost of organ transplantation and RRT. In addition, follow-up duration may be related to the survival rate after surgery, and patients with post-OLT AKI have lower survival rate than those without AKI.

Overexposure to CNI has been established as an independent factor for postoperative AKI^9^,^24^,^56^,^57^. The direct toxic effects of CNI use were the formation of thrombotic microangiopathy, such as hemolytic uremic syndrome or thrombotic thrombocytopenic purpura^24^,^57^,^58^. The severity of renal dysfunction is affected along with the change of CNI concentration. De Simone *et al*.^57^,^58^ found that everolimus with reduced tacrolimus can improve renal function after liver transplantation. In our analysis, the initial induction of CNI with tacrolimus or cyclosporine was introduced in the AKI group than the non-AKI group. A significant higher average trough level of tacrolimus was found in the AKI group than the non-AKI group. Reduced CNI levels combined with MMF use can maintain adequate immunosuppression and mitigate the incidence of post-OLT AKI. However, CNI use without combined MMF was an independent risk factor of post-OLT AKI after matching, and this result was in agreement with previous reports^24^,^32^. In addition, overexposure to CNI may also be considered as an independent factor for postoperative AKI after matching. This may be related to MMF use with reducing CNI dose in patients with high CNI trough levels, or MMF use as an initial immunosuppressive protocol. Thus, we speculate that reducing CNI dosage with MMF use can contribute to the prevention of severe AKI and should be taken into consideration in the use of nephrotoxic immunosuppressive regimens in liver transplantation^24^,^54^.

Finally, multivariate analysis revealed that the independent risk factors for the occurrence of post-OLT AKI included intraoperative blood loss, pre-SCr, cold ischemia time, warm ischemia time, treatment period with dopamine, overexposure to CNI, and combined MMF use after adjusting for other variables. Some studies^18^,^19^,^24^,^59^ identified intraoperative blood loss (>2500 mL) as an independent risk factor for the occurrence of AKI. Our results support the previous findings that blood loss could result in hypotension, unstable systemic hemodynamics, and negative influence on post-transplant renal function. However, although we define SCr > 354 μmol/L as one of the diagnostic markers of AKI (failure stage), some slight injury to the kidney (risk and injury stages) might already have existed in patients before transplantation. These slight reversible injuries progress into severe AKI (failure stage) under the influence of the instability of intraoperative systemic hemodynamics. Besides, we also confirmed that cold (>6 h) and warm ischemia times (>10 min) are independent risk factors of post-OLT AKI, which are similar to the results of Leithead *et al*.^55^, Kubal *et al*.^60^, and other studies^54^,^61^,^62^. Although grafts are separated from donors and cut down the bloodstream, cell metabolism continues, and the metabolic waste of aerobic and anaerobic metabolism cannot be eliminated, leading to cell apoptosis. Ischemia–reperfusion injury can activate cell signals linked to invasion and migration by disrupting the hepatic microcirculation. A cellular cascade can be activated by ischemia, leading to large cellular proliferation, growth, and angiogenesis^61^,^62^. As a result, the “abnormal graft” increases the burden of the kidney, resulting in AKI. Besides, ROC analysis (AUC = 0.740) and H–L test (P = 0.142) also provided evidence for the prediction of post-OLT AKI with this seven-factor model.

In summary, to mitigate postoperative AKI incidence, the following terms should at least be performed: (i) strictly monitoring the dosage of CNI and combine it with MMF duly, (ii) reducing those risk factors influenced the hemodynamic stability of patients as much as possible during and after surgery, and (iii) reducing the ischemia time of grafts.

This study has some limitations, including a not comprehensive retrospective design and the difference of diagnosis and treatment level of different centers and hospitals, which contribute to missing or absent important data. Further studies should focus on the economic level, donor factors, induction therapy, and postoperative complications to better understand the pathophysiology of post-OLT AKI and to investigate factors associated with worse outcome. Early recognition, prevention, and treatment of AKI after OLT may be useful in determining its prognosis.

In conclusion, AKI is a common complication of OLT. Blood loss, cold ischemia time, warm ischemia time, preoperative SCr level, treatment period with dopamine, overexposure to CNI, and combined MMF use are the independent risk factors of post-OLT AKI, and not MELD score, Child–Pugh grade, preoperative hypoalbuminemia, cirrhosis, hepatic failure, and duration of surgery.

## Methods

On January 6, 2016, we collected 5074 donation after cardiac death (DCD) OLT recipients who underwent surgery between January 1, 2010, and December 31, 2015. These data were obtained from 86 academic hospitals or transplant centers in mainland China and registered in a legal organ donation system, known as the China Liver Transplant Registry (CLTR)^63^, which was set up in 2005 and regulated by the Chinese government. The study was approved by the ethics committees of China Medical University and carried out in accordance with the principles of the Declaration of Helsinki as revised in 2008. All participants were supplied with oral and written information and gave written consent prior to inclusion and informed consent was obtained from all the study participants.

### Inclusion and exclusion criteria

We included all patients (>18 years) who underwent OLT between January 1, 2010, and December 31, 2015. Exclusion criteria were the following: (1) re-transplantation (n = 8); (2) RRT before OLT (n = 321); (3) combined transplantations (e.g., liver and kidney combined transplantations) (n = 175); (4) kidney transplantation before OLT (n = 19); or (5) living donor (n = 61).

### Definition of AKI

Based on the RIFLE classification^8^, AKI after OLT was classified into three groups (risk, injury, and failure) based on relative changes of SCr or urine output. We defined AKI as renal function reaching the level of failure (an increase in SCr ≥ 3.0 baseline, decrease in GFR ≥ 75%, or absolute SCr ≥ 354 μmol/L, with an acute increase of at least 44 μmol/L and/or urine output < 0.3 mL/kg/h ≥ 24 h or anuria ≥ 12 h) within their hospital stay period.

### Chinese criteria for human organ donation

Classification I of Chinese criteria (C-I): donation after brain death^64^.

Classification II of Chinese criteria (C-II): DCD, which includes classifications I–V of the Maastricht criteria^65^.

Classification III of Chinese criteria (C-III): donation after brain death awaiting cardiac death (DBCD), which is similar to classification IV of the Maastricht criteria. The warm ischemia time of donor organ is controllable.

In China, no law exists regarding identifying brain death and allowing organ donation from patients or individuals who are brain dead. Second, the family members cannot accept that organs are donated from donors whose heart is still beating. Thus, we mostly used the DBCD criteria in China. These criteria are in line with Chinese circumstances.

### Liver donor data

The following data were analyzed: age, sex, blood type, China classification of DCD^64^, cause of donor death, past medical history (including diabetes, hypertension, respiratory disease, etc.), blood components (including Na^+^, K^+^, blood urea nitrogen [BUN], albumin, alkaline phosphatase [ALP], blood sugar, total bilirubin, SCr, gamma-glutamyl transferase [GGT], aspartate aminotransferase [AST], and alanine aminotransferase [ALT]).

### Pretransplantation recipient data

The following baseline characteristics were analyzed: age, sex, body mass index (BMI), and blood type. Furthermore, we analyzed underlying liver disease (including hepatocarcinoma related, Hepatitis B, cirrhosis, hepatic failure), dialysis, total bilirubin, SCr, international normalized ratio (INR), MELD score^66^, Child–Pugh grade^67^, and albumin.

### Intraoperative data

All patients were transplanted without venovenous bypass^68^, we analyzed operative time, cold ischemia time, warm ischemia time, intraoperative blood loss, blood product transfusion (including whole blood, red blood cells, cell saver, autologous blood cell transfusion, fresh frozen plasma, and platelet), and total intravenous infusion. In addition, noradrenaline was used when the cardiac preload and systolic function were normal, but systemic vascular resistance index was low, and dobutamine was used when systolic dysfunction existed to maintain the essential hemodynamic objective (mean arterial pressure [MAP] ≥ 70 mmHg) and ensure sufficient cardiac function^3^.

### Postoperative data

We collected the following data: postoperative complications (including intraperitoneal hemorrhage, biliary complications, vascular complications, abnormal graft function, postoperative infection, diabetes, new-onset diabetes, hypertension, new-onset hypertension, cytomegalovirus infection, pleural effusion, pneumonedema, and intra-abdominal abscess), hospital day, and follow-up duration. The conditions of the use of immunosuppression, induction therapy, steroid drugs, CNIs were analyzed. The CNIs were introduced on day 0 after surgery. Outpatient follow-up was performed once a week for the first 3 months and every 2 weeks within the first year after the surgery. The causes of death for each patient were recorded. All follow-up data were collected until December 31, 2015.

### Statistical analysis

We first compared the data distribution of each covariate between the exposed and the non-exposed groups, using the Student *t* test (normal distribution) or Mann–Whitney U test (non-normal distribution) for continuous variables and *X*^*2*^ tests or Fisher’s exact test for categorical data, as appropriate. The Kaplan–Meier curves were constructed to determine survival and compared using the log rank test. A series of exact logistic regression models were implemented to obtain bivariate estimates and confidence intervals (CIs) with a small sample size. Significant predictors (P < 0.05) and only the variable that was more biologically plausible were included in subsequent multivariate model building with adjustments for covariates. Multivariate logistic regression models were used to identify whether these covariates had an independent effect on AKI after OLT. A receiver operating characteristic (ROC) curve and the goodness-of-fit test of Hosmer–Lemeshow (H–L) were used to assess the predictive power of the logistic regression model. We calculated the area under curve (AUC) and decided on a cutoff value by using the Youden method. All results were expressed as means and standard errors of the mean (SEMs). All statistical analyses were performed using SAS version 9.2 (SAS Institute Inc., Cary, NC, United States). A P-value of <0.05 was considered significant.

## Additional Information

**How to cite this article**: Zongyi, Y. *et al*. Risk factors of acute kidney injury after orthotopic liver transplantation in China. *Sci. Rep.* **7**, 41555; doi: 10.1038/srep41555 (2017).

**Publisher's note:** Springer Nature remains neutral with regard to jurisdictional claims in published maps and institutional affiliations.

## Figures and Tables

**Table 1:** Donor characteristic with or without post-OLT AKI.

Parameters (n = 4482)	Non-AKI	AKI	P value	Missing value
Age (years)	37.92 (22.63, 47.63)	39 (24, 47.17)	0.597	679
Sex (male)	3140 (81.16)	155 (81.58)	0.885	423
Blood type			0.928	388
O	1535 (39.32)	79 (41.58)		
A	1099 (28.15)	52 (27.37)		
B	994 (25.46)	47 (24.74)		
AB	276 (7.07)	12 (6.32)		
China classification of DCD			0.005	60
Grade I	536 (12.67)	19 (9.95)		
Grade II	1822 (43.06)	105 (54.97)		
Grade III	1873 (44.27)	67 (35.08)		
Cause of donor death			0.007	1765
Trauma	1452 (56.61)	86 (56.58)		
Cerebrovascular accident	1452 (56.61)	86 (56.58)		
Brain tumor	134 (5.22)	16 (10.53)		
Hypoxic brain injury	73 (2.85)	5 (3.29)		
Others	255 (9.94)	4 (2.63)		
Toxicosis	7 (0.27)	0 (0)		
Preoperative data of donor				
Past medical history				
Diabetes	8 (0.19)	0 (0)	—	
Hypertension	121 (2.82)	8 (4.12)	0.289	
Respiratory diseases	4 (0.09)	1 (0.52)	0.199	
Cardiovascular diseases	11 (0.26)	0 (0)	—	
Others	69 (1.61)	9 (4.64)	0.002	
None	1709 (39.86)	91 (46.91)	0.050	
Donor blood examination				
Na^+^	144 (138, 155)	153.15 (138.9, 164)	0.008	2810
K^+^	3.99 (3.57, 4.5)	4 (3.5, 4.6)	0.465	3040
BUN	6.83 (4.68, 10.83)	9.78 (5.41, 15.14)	<0.001	2655
Albumin	33.38 (28, 39)	30.6 (27.3, 36.6)	0.003	2652
ALP	87 (58.5, 122)	85 (61.5, 116.9)	0.825	3122
Blood sugar	7.65 (5.5, 10.9)	8.5 (6.8, 11.69)	0.039	3428
Total bilirubin (μmol/L)	14.5 (9.3, 23)	14.65 (9.6, 22.9)	0.782	2601
sCr (μmol/L)	79 (53.9, 121.95)	103.5 (64.85, 206.5)	<0.001	2598
GGT	37 (19, 79)	42 (14, 87)	0.607	3005
AST (U/L)	50.4 (30, 91.2)	61 (37, 113)	0.011	2641
ALT (U/L)	36 (21, 76)	41.25 (23.5, 75)	0.330	2624

**Table 2:** Demographic and preoperative clinical data of recipients with or without post-OLT AKI.

Characteristics	Non-AKI (n = 4288)	AKI (n = 194)	P value
Age, years (mean)	48.4 (40.4, 55.8)	50.5 (41.9, 58.7)	0.056
Male sex, n (%)	3444 (80.32)	147 (75.77)	0.121
BMI, (mean, SD)	22.77 (20.76, 24.69)	22.33 (20.76, 24.49)	0.072
Preoperative data, n (%)			
Blood type compatibility, n (%)	3757 (87.62)	175 (90.21)	0.004
Hepatocarcinoma-related disease, n (%)			<0.001
HCC	1630 (38.01)	54 (27.84)	
Benign disease	1630 (38.01)	68 (35.05)	
Others	1028 (23.97)	72 (37.11)	
Hepatitis B, n (%)	2723 (63.5)	130 (67.01)	0.321
Cirrhosis, n (%)	3411 (79.55)	138 (71.13)	0.005
Hepatic failure, n (%)	311 (7.25)	35 (18.04)	<0.001
Preoperative dialysis, n (%)	312 (7.28)	20 (10.31)	0.115
Total bilirubin (μmol/L), (mean L b)	102 (27.8, 356)	143 (29, 450)	0.461
Serum creatinine (μmol/L), (mean L c)	74.6 (56.2, 110)	85 (64, 136)	0.003
INR, (mean score)	1.58 (1.2, 2.62)	1.63 (1.25, 2.5)	0.485
MELD score, (mean score)	20 (12, 33)	23 (13, 35)	0.109
Child-Pugh grade, (mean C-P)	10 (8, 12)	10 (8, 12)	0.204
Albumin (g/dL), (mean min)	3.34 (2.91, 3.8)	3.38 (3, 3.89)	0.571

**Table 3:** Intraoperative clinic data (mean range) of recipients with or without post-OLT AKI.

Parameters	Non-AKI (n = 4288)	AKI (n = 194)	P value
Duration of surgery (h)	7.25 (6.07, 8.53)	7.28 (5.67, 8.92)	0.916
Cold ischemia time (h)	6.17 (4.5, 8.63)	7 (5, 10.53)	0.043
Warm ischemia time (min)	5 (2, 8)	5 (3.25, 14)	<0.001
Intraoperative blood loss (mL)	1600 (800, 3000)	2500 (1500, 6000)	<0.001
**Transfusion of blood products**			
Whole blood (mL)	1200 (800, 2400)	1260 (800, 2000)	0.627
Red blood cells (mL)	1600 (800, 2400)	2000 (1100, 4000)	<0.001
Cell saver (mL)	2800 (1600, 4000)	4600 (800, 6400)	0.701
Autologous blood cell transfusion (mL)	800 (500, 1500)	1225 (600, 2950)	0.015
Fresh frozen plasma (mL)	1450 (800, 2060)	1850 (1080, 2810)	<0.001
Platelet (units)	2 (1, 10)	2 (1, 9)	0.764
Total volume of intravenous infusion (mL)	5500 (3500, 7652)	6611.5 (4975, 9990)	<0.001
Noradrenaline (%)	1458 (34)	107 (55.2)	<0.001
Dobutamine (%)	772 (18)	72 (37.1)	<0.001

**Table 4:** Postoperative clinical data of recipients with or without post-OLT AKI.

Factors	Non-AKI (n = 4288) (%)	AKI (n = 194) (%)	P value
Postoperative complications			
Intraperitoneal hemorrhage	194 (4.52)	64 (32.99)	<0.001
Biliary complications	198 (4.62)	10 (5.15)	0.728
Vascular complications	152 (3.54)	17 (8.76)	<0.001
Abnormal graft function	71 (1.66)	55 (28.35)	<0.001
Postoperative infection	677 (15.79)	87 (44.85)	<.001
Diabetes	537 (12.52)	42 (21.65)	<0.001
Hypertension	144 (3.36)	12 (6.19)	0.036
New-onset diabetes	430 (10.03)	32 (16.49)	0.004
New-onset hypertension	94 (2.19)	6 (3.09)	0.406
Cytomegalovirus infection	51 (1.19)	2 (1.03)	0.847
Pleural effusion	989 (23.06)	95 (48.97)	<0.001
Pneumonedema	40 (0.93)	20 (10.31)	<0.001
Intra-abdominal abscess	481 (11.22)	64 (32.99)	<0.001
Initial induction of CNI			
Tacrolimus	2680 (62.51)	146 (75.02)	0.015
Cyclosporine	391 (9.11)	25 (13.04)	0.027
Average CNI trough level (ng/ml)			
Tacrolimus	9.2 (8.8, 9.6)	10.3 (9.8, 10.7)	0.008
Cyclosporine	178.5 (172, 185)	174.1 (162, 181)	0.324
Overexposure to CNI	527 (12.30)	93 (48.14)	<0.001
MMF use	3044 (71.08)	81 (42.10)	<0.001
Dobutamine (days)	1.2 (0.0, 2.4)	3.5 (0.8, 6.2)	<0.001
Dopamine (days)	3.2 (1.1, 5.3)	5.4 (3.5, 7.3)	<0.001
Length of hospital stay (days)	25 (18,35)	16 (6,34.5)	<0.001
Follow-up duration (month)	3.82 (0.92, 10.66)	0.72 (0.2, 3.88)	<0.001

**Table 5:** Multivariate analysis of the risk factors of post-OLT AKI.

Factors		OR	95% CI		P value
Cold ischemia time	<7 h	1	—		—
	>7 h	1.061	1.032	1.09	<0.001
Warm ischemia time	<10 min	1	—		—
	>10 min	1.028	1.011	1.046	0.001
Blood loss	<2500 mL	1	—		—
	>2500 mL	1.23	1.001	1.451	<0.001
SCr	<354 μmol/L	1	—		—
	>354 μmol/L	1.352	1.181	1.763	<0.001
Treatment with dopamine	<6 days	1	—		—
	>6 days	1.854	1.425	2.281	<0.001
Overexposure to CNI	No	1	—		—
	Yes	2.841	1.762	5.360	<0.001
Combined use of MMF	Yes	1	—		—
	No	2.184	1.338	6.89	0.023
